# On the origin of Earth’s Universal BioConsciousness

**DOI:** 10.3389/fpsyg.2025.1636473

**Published:** 2026-02-09

**Authors:** Akbota Tleuberdinova

**Affiliations:** Independent Researcher, University of Toronto, Toronto, ON, Canada

**Keywords:** FUCA, evolution, consciousness, thought, language, cell, DNA, artificial intelligence

## Abstract

The origin of consciousness remains one of the oldest problems in both science and philosophy. Several emerging theories provide new perspectives on the origin and evolution of consciousness, including Cellular Basis of Consciousness (CBC) theory and the Cognition-Based Evolution (CBE) theory. Moreover, declarations such as the New York Declaration on animal consciousness further underscore the need for a unified understanding of consciousness. To bridge this gap, this paper presents a unified framework on the origin of Earth Universal BioConsciousness through the intersection of evolutionary biology, consciousness studies, and philosophy, incorporating the following core principles: (1) Universal BioConsciousness (UBC) theory or BioConsciousness originates from BioConsciousness (BoB) theory states that cell bioconsciousness originates from pre-existing cell bioconsciousness, developing not spontaneously but rather continuously from pre-existing first conscious cell—FUCA; (2) Absolute BioConsciousness and Relative Cell Biomatter (ABC) theory. suggests that bioconsciousness evolution precedes genome and organic evolution: (2.1) Spencer’s theory of life states that life precedes organization; (2.2) Minot’s theory of biological consciousness is the primary cause of biological evolution; (2.3) Popper’s theory of evolutionary hierarchy: functional changes precede structural ones, and evolution proceeds as a cyclical process; (3) Universal Genome Evolution (UGE) theory states that universal DNA is a structural correlate of universal bioconsciousness; in this framework, bioconsciousness is a primary causal agent, directing genetic adaptations; (4) Ontogenetic Evolution of Universal Bioconsciousness (OEUB) theory states that the individual lifetime’s evolution of bioconsciousness begins at the moment of egg cell fertilization—zygote; furthermore, (5) The first principle of philosopher Descartes, ‘Cogito ergo sum,’ gains a novel interpretation through the exploration of relationships between bioconsciousness, verbal thought, and verbal language. Consequently, the (6) Verbal Communication Sense (VCS) theory argues that verbal thought is dependent on bioconsciousness and lacks an independent capacity for thinking; therefore, its primary function is ”communication sense’’. 7) Positive and Negative BioConsciousness (PNB) theory suggests that unicellular bioconsciousness and multicellular bioconsciousness exist in two forms: positive and negative. Finally, (8) the emergence of human-like artificial consciousness is highly unlikely, as bioconsciousness is tied to biological lineage and does not arise spontaneously.

## 1 Introduction

“What would it mean if we actually did discover the ultimate theory of the universe? It would be the ultimate triumph of human reason—for then we would know the *Mind of God*”. (Hawking, 1988)

“The new theory implies a change in the rules governing the prior practice of normal science. Its assimilation requires the reconstruction of prior theory and the re-evaluation of prior fact.” Kuhn (1962)

Inspired by Hawking’s original quote, this work will explore the question: What would it mean if we actually discovered a unified theory of consciousness? (Hawking, 1988). With artificial intelligence’s rapid progress, bridging gaps in understanding consciousness is an urgent scientific problem, which, if solved, will have a profound impact across several domains, including science, medicine, animal welfare, plant biodiversity, law, and technology (Cleeremans et al., 2025). This paper supports the notion that consciousness is not merely a byproduct of the brain but the original signature of life itself. The origin of bioconsciousness can be understood through an the evolutionary biology and cellular perspective, as it is a dynamic and continually evolving process that manifests itself in the increasing biological complexity of living organisms (Veit, 2022). For nearly 170 years, the most dominant theory of evolution has been Darwin’s theory of evolution by natural selection and genetic mutation, which disproved the notion of the fixity of species, as also outlined in Lamarck’s theory of descent (Lamarck, 1809; Darwin, 1859).

Recently, researchers have concluded that consciousness is a fundamental property of every living being, from the first cells to complex living organisms (Reber, 2019; Reber et al., 2023; Miller, 2023; Cooke, 2020; Baluška, 2025). Emerging theories on cellular-based consciousness and its role in evolution are sparking new debates on the biological perspective of consciousness and its integration into conventional evolutionary theories (Mallatt et al., 2021; Robinson et al., 2024; Andrews et al., 2024). To address this gap, this paper presents a unified perspective on the origin of Earth’s Universal BioConsciousness by integrating biological evolution theories with novel theories of cellular-based consciousness (Baluska, 2025; Reber, 2019; Reber et al., 2023; Miller, 2023; Cooke, 2020). This article is divided into two main parts: the first, *on the Origin of Earth Universal Bio-Consciousness*, presents a unified framework for understanding the origin of universal bioconsciousness and its primary role in biological evolution; the second, *Search After the Truth*, presents Descartes’ first principle, “Cogito ergo sum,” with a novel argument and interpretation, distinguishing between bioconsciousness, verbal thought, and verbal language.

## 2 Part 1: On the Origin of Earth’s Universal BioConsciousness

“Although our understanding of the evolutionary emergence of the very first cells is obscured by the extremely long timeline since that revolutionary event, *the generally accepted position is that the de novo formation of cells is not possible*; all present cells are products of other prior cells. All currently living cells have direct structural and functional connections to the very first cells. All cells are endowed with individual sentient cognition that guides their individual agency, behaviour and evolution.” (Baluška et al., 2023)

“What the Cellular Basis of Consciousness (CBC) stance maintains is that sentience, cognition, and consciousness (whatever term you wish) followed the same evolutionary path. Those ancient prokaryotes were sentient and all the species that evolved from their original biomolecular platform were and are sentient. We anticipate that this perspective will continue to attract attention within the field and that, as more researchers pursue the entailments of the CBC, insights and understanding will follow. We look forward to a paradigm shift in evolutionary biology.”

Recent studies concluded that all cells are conscious (Reber, 2019; Miller, 2023; Baluška, 2025; Shapiro, 2021). Although the origin of life is unknown, researchers have concluded that the first cell, FUCA, could be the starting point of life (BioConsciousness) on Earth (Prosdocimi et al., 2019; Forterre, 2015). Therefore, we suggest that the first life (bioconsciousness) emerged with the first proto-cells, such as FUCA and LUCA, as depicted in the *Tree of Life* (Prosdocimi et al., 2019; Forterre, 2015). This paper supports the notion that after originating in the first FUCA cell, bioconsciousness thereafter cannot arise spontaneously, but only through uninterrupted, continuous evolution by cellular division of that first conscious cell. Consequently, the question of how the first FUCA’s bioconsciousness originally emerged is difficult to answer, given the limited scientific evidence. However, in recent study examining the origin of life, authors concluded that life begins in the self-replicating RNA-dominated context, which known as the RNA World hypothesis (Fine and Moses, 2025)

“Intriguingly, a *spontaneously forming condensate* capable of template-based hydrolysis of RNA polymerization provides (in principle) a mechanism for both compartmentalization and *self-replication* (increasingly probable caused by bioconsciousness added by author). In other words, we contemplate the possibility that the condensate itself was a *self-replicative* catalyst during the *origin of life*. Once the condensate breaks apart, the ‘offspring’ condensates can undergo further *growth and division*, resulting in a positive feedback loop that produces a ‘*condensate chain reaction*’.” (Fine and Moses, 2025)

**Figure 2:**
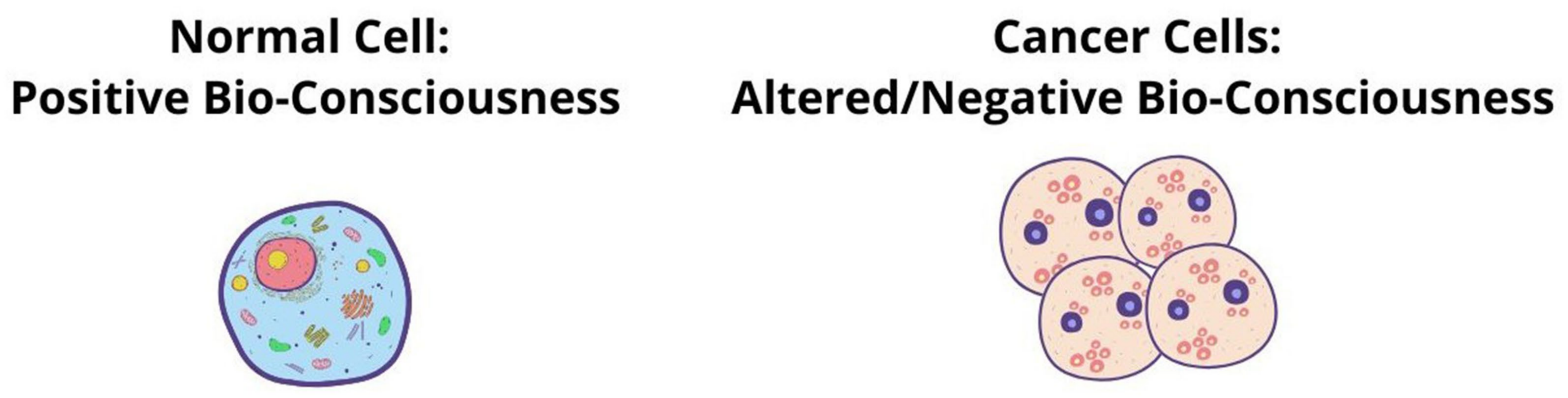
Absolute BioConsciousness and Relative Cell Biomatter (ABC) theory.

The authors acknowledge that after initial spontaneous origin: “RNA condensates may spontaneously begin”, thereafter the RNA condensate “can undergo further growth and division,” as depicted in Figures 1, 2: “proposed mechanism of compartmentalization and self-replication via an RNA condensate: (1) condensation, (2) RNA-templated polymerization and growth, and (3) division (*self-replication*)” (Fine and Moses, 2025). To fully understand the possibility of *abiogenesis* (Kipping, 2025), in other words the spontaneous generation of bioconsciousness, we should exclude the concept of “panpsychism” (consciousness is always present, “proto-mental”) (Jarocki, 2023), which is the endeavor of future scientific investigations.

**Figere 1:**
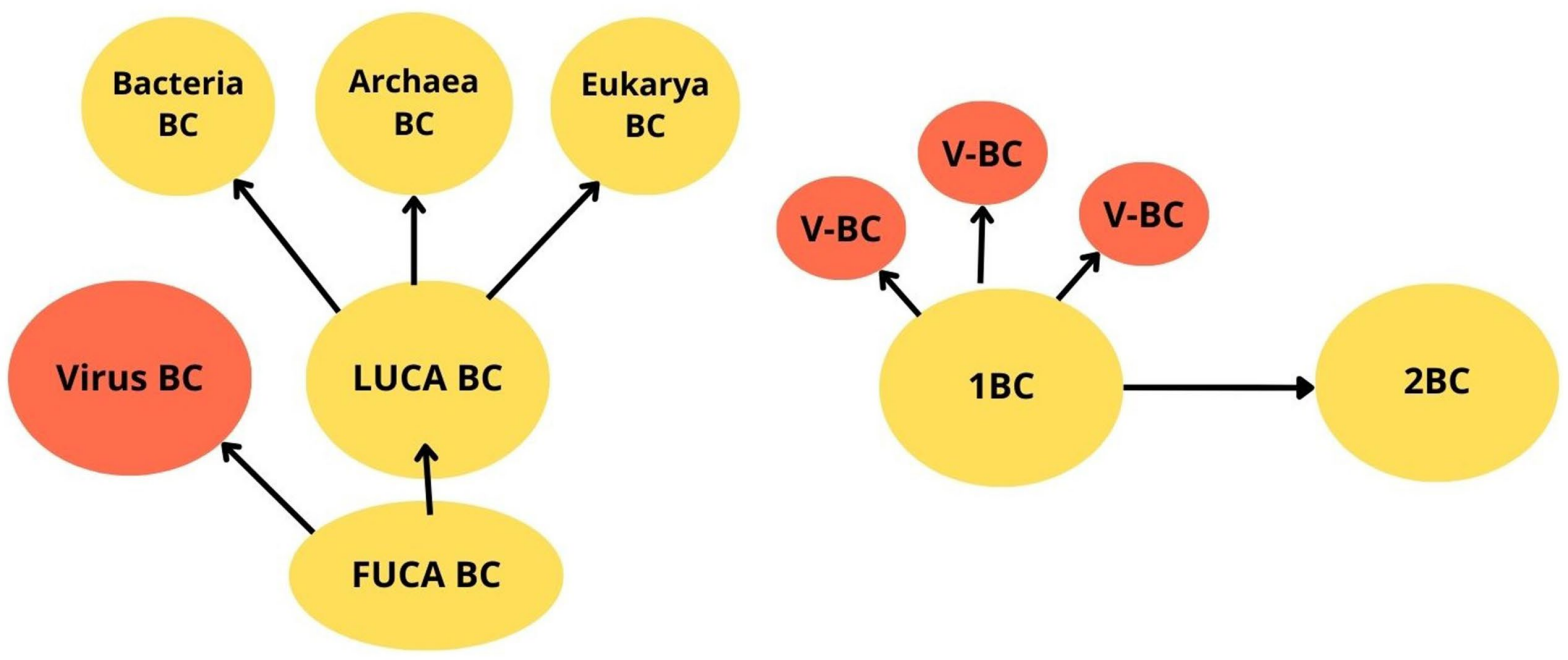
Evolution of Earth’s Universal BioConsciousness. FUCA, the first universal common ancestor cell; LUCA, the last universal common ancestor cell; BC, bioconsciousness; V, virus.

Panpsychism is a metaphysical view according to which at least some of the properties constituting the fundamental level of reality are mental or proto-mental. (Jarocki, 2023)

*Due to our cognitive limitations*, we do not know—and probably never will know—the nature of intrinsic properties of the ultimate stuff in which the *physical world is grounded*. If this is true, then the standoff between materialism and panpsychism will perhaps be *impossible to solve*, leaving us in a state of *permanent agnosticism* about the nature of the constituents of reality. (Jarocki, 2023)

In this paper, I assume that after the initial origin, highly probable in RNA world through RNA condensates principle (Fine and Moses, 2025), the bioconsciousness undergoes the uninterrupted growth and division by *self-replication*, which further lead to formation of first proto cells, such as FUCA and LUCA (Prosdocimi et al., 2019; Forterre, 2015).

### 2.1 Universal BioConsciousness (UBC) theory or BioConsciousness originates from BioConsciousness (BoB) theory

“Nothing in biology and evolution makes sense except in the light of cognition.” (Miller, 2023, p. 1)

“Gases, fluids, electricity, magnetism, ozone, things known or things occult, there is nothing in the air that is conditional to life, except the germs that it carries; for life is a germ and a germ is life. Never will the doctrine of *spontaneous generation* recover from the mortal blow of this simple experiment.” Louis Pasteur, 1859 (Vallery-Radot, 1911, pp. 94–109)

“Now the liquid of this second flask will remain pure not only two days, a month, a year, but three or four years—for the experiment I am telling you about is already four years old, and the liquid remains as limpid as distilled water.” Louis Pasteur, 1859 (Vallery-Radot, 1911, p. 108)

In contrast to conventional theories of consciousness (Mallatt et al., 2021; Robinson et al., 2024), a growing body of evidence suggests that bioconsciousness is not exclusively a function of the nervous system (Reber et al., 2023; Miller, 2023; Cooke, 2024). Recent advancements in the field of consciousness research have increasingly supported the notion that life and consciousness are intrinsically connected. These findings suggest that consciousness originated with the first unicellular organisms, such as prokaryotic cells, around 3.8 billion years ago, marking it as a fundamental attribute of life itself (Baluška and Reber, 2019, 2021; Baluška et al., 2023; Miller, 2023; Reber, 2019; Reber et al., 2023). This section explores the evolution of Earth bioconsciousness (EBC), tracing its development from the earliest manifestation of bioconsciousness found in the first universal common ancestor (FUCA) cell. FUCA came before LUCA, forming when self-replicating RNA-like molecules started bind aminoacids in a world without oxygen. (Prosdocimi et al., 2019). The LUCA originates from the pre-LUCA evolution of the first universal cellular ancestor cell (FUCA), which gave rise to the three domains of life: bacteria, archaea, and eukarya (Prosdocimi et al., 2019; Forterre, 2015).

The core principle of the cellular basis of consciousness (CBC) theory, as proposed by Reber et al. (2023), is that life and sentience (cell bioconsciousness) are inseparable, meaning that wherever there is life, there is also consciousness. Once cognition, communication, and problem-solving became integrated within cellular life, the continued development of life can be understood as a series of self-similar reproductions (Reber et al., 2023). Therefore, cellular cognition and sentience (cell bioconsciousness) are a fundamental drive behind all biological and evolutionary progression (Reber et al., 2023). The functions of life are defined by Reber as follows: (1) *self-replication*; (2) metabolism; (3) external cell membrane; (4) sentience (*cell bioconsciousness*)—the mental subjective part is essential for the survival of every cell (Reber, 2019, p. 133). The living mirror theory of consciousness arrives at the same conclusion: (Cooke, 2024, p. ix). This theory proposes that all life is conscious, and multiple conscious systems exist within complex life forms (Cooke, 2020). Additionally, this theory posits that phenomenal consciousness is a consequence of anti-entropic dynamics, a defining feature of all life (Cooke, 2020; Cooke, 2021). At the informational level, these entropy-resisting dynamics produce “an internal framework of beliefs in survival-relevant qualities in the outside world and that this framework of beliefs is an equivalent description of consciousness itself” (Cooke, 2020).

Cognition-Based Evolution (CBE) theory posits that consciousness is an intrinsic part of all living forms, as an integrated form of information (Miller, 2023). Any cell can perform an internal analysis of information, demonstrating that it possesses intelligence and memory (Miller, 2023, p. 198). According to Miller, the integration system of consciousness is a combined product of “the individual “state” function of consciousness of each of our constituent cells, including the contribution from our *holobionic microbial partners* (Miller, 2023, p. 204). Furthermore, the New York declaration on Animal Consciousness, signed by more than 572 researchers worldwide, states that “a very wide range of animals, including all vertebrates and many invertebrates, can have subjective experiences” (Andrews et al., 2024). From the aforementioned theories, we can conclude that bioconsciousness is an intrinsic property of life, existing inseparably and simultaneously throughout evolution; thus, life and bioconsciousness are coterminous, evolving together as an unbroken continuum.

The idea of spontaneous generation of life was disproved by Louis Pasteur in 1859, when he demonstrated that the second flask, after being boiled to destroy any germs, remained unchanged for 4 years (Vallery-Radot, 1911, pp. 108–109). Moreover, this idea was confirmed by Rudolf Virchow, who formulated the principle “Omnis cellula e cellula”—meaning *every cell originates from a preexisting cell* through cell division. His work established that life is sustained through continuous cellular reproduction, reinforcing the concept of uninterrupted biological and conscious development (Virchow and Chance, 1858). Therefore, as life and bioconsciousness are inseparable, we can suggest that the bioconsciousness of every cell originates from a preexisting bioconsciousness of the parent cell by cell division.

Universal BioConsciousness (UBC) theory or BioConsciousness originates from BioConsciousness (BoB) theory proposes that bioconsciousness originated from the first FUCA cell and has undergone uninterrupted and ongoing progression, evolving alongside biological life to encompass all biological life on Earth. In other words, it suggests that life-consciousness originates from pre-existing life-consciousness rather than spontaneously arising (exception: the initial origin of bioconsciousness, which is unknown), and bioconsciousness has evolved continuously and uninterruptedly through cellular division, extending from FUCA to all its biological descendants of the three domains of life: bacteria, archaea, and eukarya (Figure 1). After the initial origin, bioconsciousness cannot emerge spontaneously thereafter, but evolves uninterruptedly and continuously by cell division, following the pattern: 1BC → 2BC, where one parent cell bioconsciousness (1BC) divides into two offspring cell bioconsciousness (2BC) (Figure 1). There is an ongoing debate about whether viruses are living organisms or nonliving organisms (Prosdocimi et al., 2019; Damasio, 2021, pp. 39–40). According to the BoB theory, the virus replicates itself from another “host” cell bioconsciousness, which implies that the virus possesses life-consciousness. Thus, viruses do not arise spontaneously, but only from pre-existing bioconsciousness, providing clear evidence of their living nature (Figure 1).

The Evolution of Earth’s BioConsciousness (EBC) represents the collective sum of all individual units of bioconsciousness—*each living organism on the planet*—which evolved from the first FUCA bioconsciousness by cell division: one parent unit of bioconsciousness (1BC) divides into two identical daughter units of bioconsciousness (2BC) following the pattern: 1BC → 2BC (Figure 1). Bio-consciousness comes in *unicellular-unit* (primary consciousness) and “multicellular-unit” (higher-order of evolution of BC), which everyone possesses its own subjective *qualia* experience, representing a distinct form of private or first-person conscious knowledge, yet existing at different stages of progressive evolutionary development. In conclusion, universal bioconsciousness has evolved from its first ancestor, FUCA bioconsciousness, to encompass all three domains of biological life-consciousness on Earth. Further scientific experiments are required to define *clear operational criteria* such as minimal thresholds for integrated information or demonstrable behavioural correlates, to understand the mechanism of bioconsciousness at the cellular level. For example, specifying markers of cell-level consciousness, or measurable health outcomes linked to hypothesized states discussed in this paper and generating falsifiable scientific statements based on these theories.

#### 2.1.1 Terminology

In this paper the following concepts are used with the same meaning: consciousness, bioconsciousness ife-consciousness and sentienc (cell bioconsciousness or primary bioconsciousness).

### 2.2 Absolute BioConsciousness and Relative Cell Biomatter (ABC) theory

Absolute BioConsciousness and Relative Cell Biomatter (ABC) theory posits that the evolution of bioconsciousness is primary and underlies the evolution of organic biomatter. The philosopher Descartes states that “all things… where we compare them to each other… can be absolute or relative (Box 1). Thus, bioconsciousness is ’absolute’—independent, causal, universal, unitary and bio-matter is ’relative’—dependent, resultant, compound, particular, multiple, and is secondary in that it can be traced back to the absolute. In this view, cell biomatter does not generate consciousness; rather, it is its expression—a secondary and dependent phenomenon; thus, cell biomatter arises from cell bioconsciousness, not the reverse. Although all diseases have their origins in pathological changes within cells (Virchow and Chance, 1858), our theory proposes that the cell biomatter of a cancer cell is a result of the dysfunction of cell bioconsciousness (a causal element), so its structure or cell biomatter does not have its own causality. In other words, patients have cancer disease not because of cell biomatter, which is resultant, but because of dysfunction of cell bioconsciousness, which manifests itself in cancer cells (Figure 2). To conclude, the ABC theory asserts that bioconsciousness is primary and causal, while cell biomatter is secondary and dependent on bioconsciousness. This suggests that cell biostructure are shaped by bioconsciousness, rather than the other way around.

BOX 1 Rule VI “All things…that is, where we do not consider their natures in isolation, but where we compare them to each other in order to deduce some from others, may be said to be either absolute or relative. I call “absolute” whatever contains in itself the pure and simple essence with which are concerned; such as all which is considered as independent, causal, simple, universal, unitary, equal, similar, straight, or as having other qualities of this sort; and I call “the absolute” itself that which is the simplest and clearest of all, and which we can therefore use in solving further problems. And the “relative” is that which, while having the same nature or at least participating in it to some degree, is secondary in that it can be traced back to the absolute, and deduced from it by some chain of reasoning. But, in addition, it involves in its conception certain other things which I call “relations,” such as whatever is said to be dependent, resultant, compound, particular, multiple, unequal, dissimilar, oblique, and the like. And these relative things are further removed from the absolute. We are warned in this rule that all these things should be distinguished, and that their connections with one another and the natural order among them should be observed, so that we can pass from the last to the most absolute.” (Descartes, 1961, pp. 20–21)

“To achieve a full understanding of the diseases which plague humanity, we must look to cells… An understanding of all cells as sentient and cognitive living systems is essential not only for our understanding of life as a whole but also for dealing with diseases that have their roots in cellular physiology.” (Baluška et al., 2023)

In recent years, the concept of bioconsciousness-driven evolution was outlined in many publications, including in paper “Unlimited Associative Learning and the Origins of Consciousness (Birch et al., 2020), where authors proposed a new theory of evolutionary marker of consciousness as the capacity for unlimited associative learning (UAL): open-ended learning and goal-directed behaviour that drives evolution (Birch et al., 2020; Ginsburg and Jablonka, 2019). Furthermore, the following biological theories support the ABC theory: (1) Herbert Spencer’s theory of life, which asserts that life precedes organization, implying that the organizing principle (i.e., bioconsciousness) gives rise to physical form (biostructure); (2) Charles S. Minot’s theory of biological consciousness: positions consciousness as the primary driver of biological evolution, shaping form and function from within; (3) Karl Popper’s evolutionary hierarchy theory suggests that functional evolution precedes structural development, framing evolution as a cyclical process wherein inner experience molds outer embodiment.

#### 2.2.1 Herbert Spencer’s theory of life

**Figure 3:**
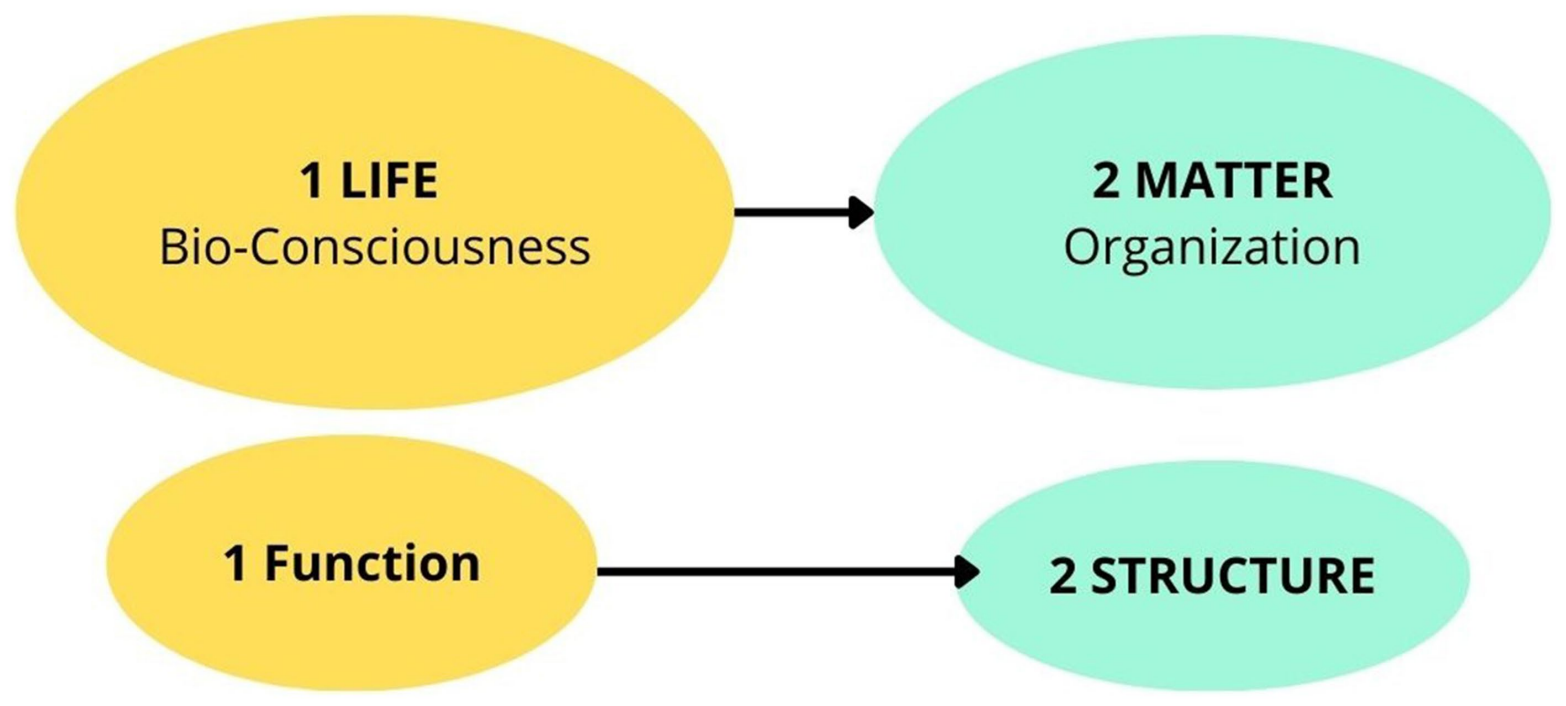
Herbert Spencer’s theory of life.

“It may be argued that on the *hypothesis of evolution*, life necessarily comes before organization. On this hypothesis, organic matter in a state of homogeneous aggregation must precede organic matter in a state of heterogeneous aggregation. But since the passing from a structureless state to a structured state is itself a vital process, it follows that vital activity must have existed while there was yet no structure: *structure could not else arise*.” (Spencer, 1898, p. 210; Figure 3).

“*That function takes precedence of structure*, seems also implied in the definition of life. If life (bioconsciousness, added by author) is shown by inner actions so adjusted as to balance outer actions—if the implied energy is the substance of life while the adjustment of the actions constitutes its form; then may we not say that the actions to be formed must come before that which forms them—that the continuous change which is the basis of function, must come before the structure which brings function into shape.” (Spencer, 1898, p. 211; Figure 3).

#### 2.2.2 Charles S. Minot’s theory of biological consciousness

“Consciousness ought to be regarded as a biological phenomenon” (Minot, 1902)

“What is the relation of *the mind to the body*? No possibility of avoiding the conclusion that consciousness stands in immediate causal relations with physiological processes; conscious actions are the primary, commanding factor of adjustment.” (Minot, 1902, pp. 8–12)

Charles S. Minot, a well-known anatomist and embryologist, proposed a definition of biological function of consciousness in the paper “*The problem of consciousness in its biological aspects*” (Minot, 1902). There were two schools of thought in the biological study of consciousness: the first school believed that consciousness is a real phenomenon, while the second school explained consciousness as an epiphenomenon, meaning it accompanies the real phenomenon but has no causal effect on physiological processes (Minot, 1902, p. 2). Minot refused to accept the epiphenomenal explanation of consciousness and believed it to be a fundamental biological phenomenon (Minot, 1902, p. 3). His main question was: “*Is consciousness a true cause*?” (Minot, 1902, p. 2).

**Figure 4:**
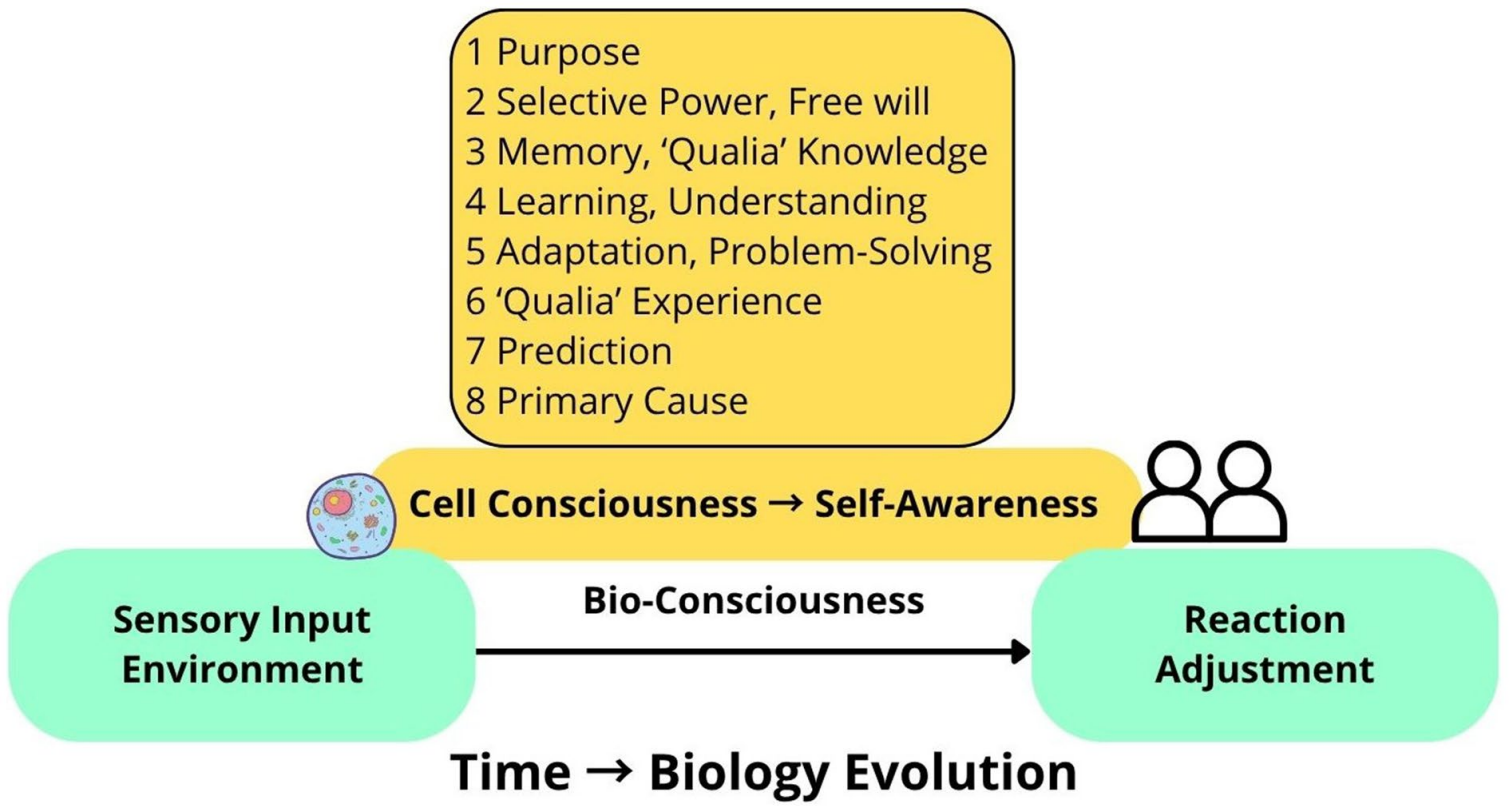
Minot’s theory of biological consciousness. “The function of consciousness is to dislocate in time the reactions from sensations” (Minot, 1902, p. 4).

He defined essential functions of consciousness in biological aspects as follows (Minot, 1902, pp. 4–6):

1. *Purpose-driven* (goal directed) is a prominent distinction between living organisms and non-living bodies, which means that any biologist can explain why a given structure or function exists; therefore, he asked a following question: “what the essential function is which it performs?” (Minot, 1902, p. 4). He defined a statement from the bionomics perspective: “The function of consciousness is to dislocate in time the reactions from sensations” (Minot, 1902, p. 4). This explanation can be applied from a biological perspective to every cell, every organ, and the entire organism, as consciousness is situated between sensory input and reaction (Figure 4).
2. *Selective power* (free will) determines the two types of conscious intervention: stopping the response (conscious inhibition) and executing a reaction (Minot, 1902, p. 5).
3. *Memory or qualia knowledge*: consciousness can combine immediate sensory input with previously memorized sensations to make more informed environmental adjustments. (Minot, 1902, pp. 5–6)
4. *Learning*: consciousness allows us to learn from past experiences. (Minot, 1902, p. 5)
5. *Adaptation* (problem-solving): “Consciousness is a device to secure better adjustment to external reality.” (Minot, 1902, p. 7)
6. *Qualia experience*: he distinguished objective reality from subjective experience: we use labels like colours to get information from external reality, but “objectively red, yellow and green do not exist.” (Minot, 1902, p. 6)
7. *Prediction*: consciousness can foresee the consequences of the organism’s responses for continual adaptation. (Minot, 1902, p. 6).
8. *Primary in evolution*: consciousness plays a leading role in the evolutionary process, and “conscious actions are primary, commanding factor of adjustment; conscious actions have been transformed into reflexes and instincts; the entire evolution of plants and animals is essentially the evolution of the means of adjustment of the organism to external conditions” (Minot, 1902).

Dr. Minot concluded:

Our sensory, motor and other organs are the servants of consciousness; its messengers or scouts; its agents or laborers; and the nervous system is its administrative office. A large part of our anatomical characteristics exist for *the purpose of increasing the resources of consciousness*. Our eyes, ears, taste, etc., are valuable, because they supply consciousness with data; our nerves, muscles, bones, etc., are valuable, because they enable consciousness to do the needed reactions; both the senses once evolved are improved and also new senses are added. It seems inevitable therefore to admit that consciousness extends far down through the animal kingdom, certainly at least as far down as there are animals with sense organs or even the most rudimentary nervous system. The development and improvement of consciousness has been the most important, really the dominant, factor in evolution. Human evolution is the continuation of animal evolution, and in both, the dominant factor has been the increase of the resources available for consciousness. (Minot, 1902, pp. 7–10)

In conclusion, biological consciousness is a complex phenomenon, which includes such functions as purpose, selective power, memory, learning, adaptation, qualia subjective knowledge, prediction, and it is a primary causal factor of biological evolution. Thus, bioconsciousness evolution is a progressive development of its function and the biological sensory structures that support it, gradually transforming from cell basic type of bioconsciousness (or primary bioconsciousness) → to self-awareness: higher types of bioconsciousness (Robinson et al., 2024).

#### 2.2.3 Karl Popper’s evolutionary hierarchy theory

“My theory may also be presented like this: higher forms arise through the primary hierarchy of *p → s → a*, that is, whenever and as long as the preference is in the lead. It is indeed the preferences which lead the way. This emphasis on preferences in my theory is, clearly, a purely “objective” affair: we need not assume that these preferences are conscious. But they may *become conscious*. My approach, therefore, leads almost necessarily to a research program that asks for an explanation, in objective biological terms, of the emergence of states of consciousness.” (Popper, 2010, pp. 172–174)

As we discussed in the previous section, bioconsciousness is the primary cause of evolution, and I will elaborate on this concept further by introducing a Popper theory of Evolutionary Hierarchy. Karl Popper, in the paper “*Darwinism as a metaphysical research programme*,” introduced an orthogenesis theory for the enrichment of Darwin’s theory of evolution by natural selection (Darwin, 1859; Popper, 2010, p. 167). “Orthogenetic trends are sequences of evolutionary changes in the same direction: nonrandom changes” (Popper, 2010, p. 170).

**Figure 5:**
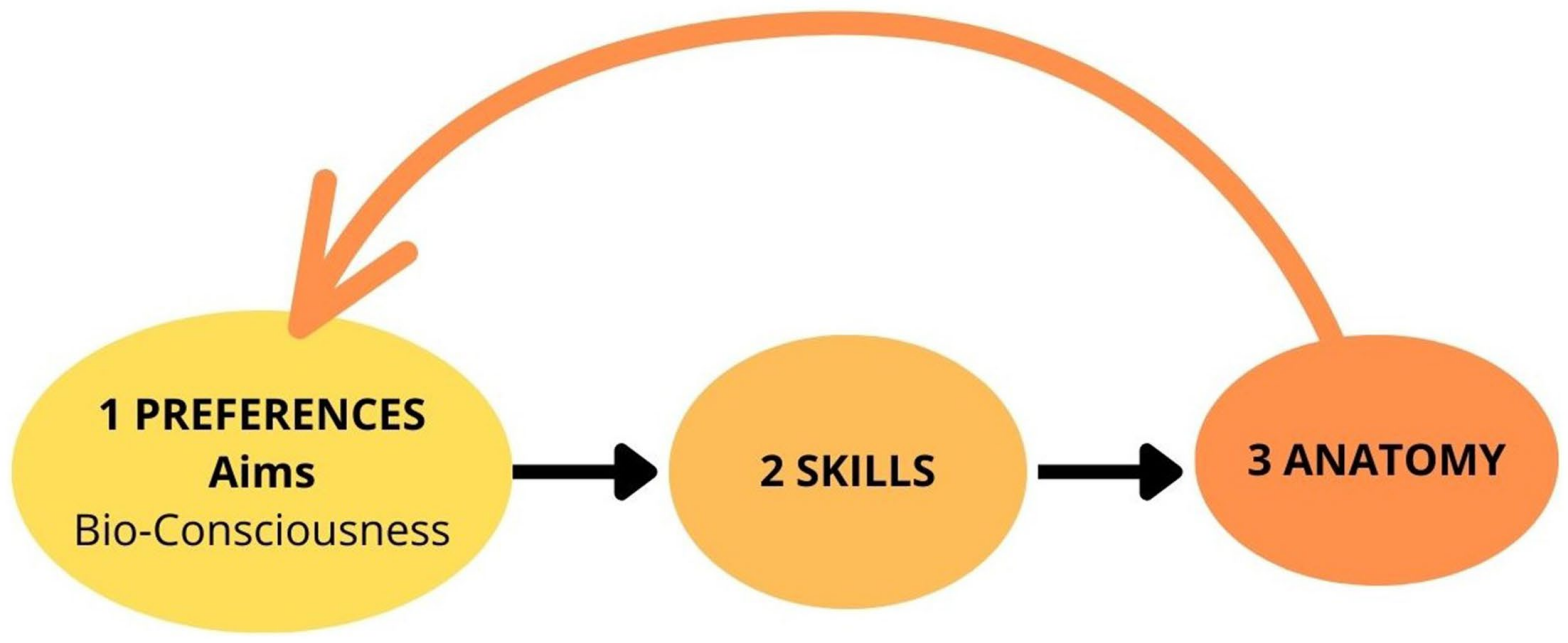
Popper’s theory of evolutionary cycle (Popper, 1974).

Popper differentiated two types of selection: external selection (environmental); and internal selection, which comes from the organism’s preferences or aims in response to external selection (Popper, 2010, p. 170). He supposed three types of gene control: A-genes control anatomy; B-genes control behaviour, which is subdivided into (1) P-genes control preferences or aims and (2) S-genes control skills (Popper, 2010, p. 170). He assumed that B-genes are responsible for pure behavioural changes in response to external selection, which means there are no hereditary anatomical changes in the genome system of the organism. For example, food disappearance (environmental change) creates a new preference or aim to adapt to a new situation, leading to a new behaviour without genetic change. If this pure behaviour adaptation is successful, it will be fixed by P-genes as a new pattern of behaviour. Thereafter, new skills for obtaining food will evolve in response to preference changes; Popper emphasized that only after the S-structure change will the A-structure change or anatomical improvement occur, so anatomical change comes last. Popper concluded that there is an evolutionary direction as follows: P → S → A. This schema can be explained as follows: ( first) preference change, (second) skill change, which further leads to (third) anatomical change. Furthermore, he suggested that this sequence could be cyclical, which could be described as the principle of mutual reinforcement: *new anatomical change will further influence preference changes* ( bioconsciousness evolution, added by author), and so on (Figure 5).

**Figure 6:**
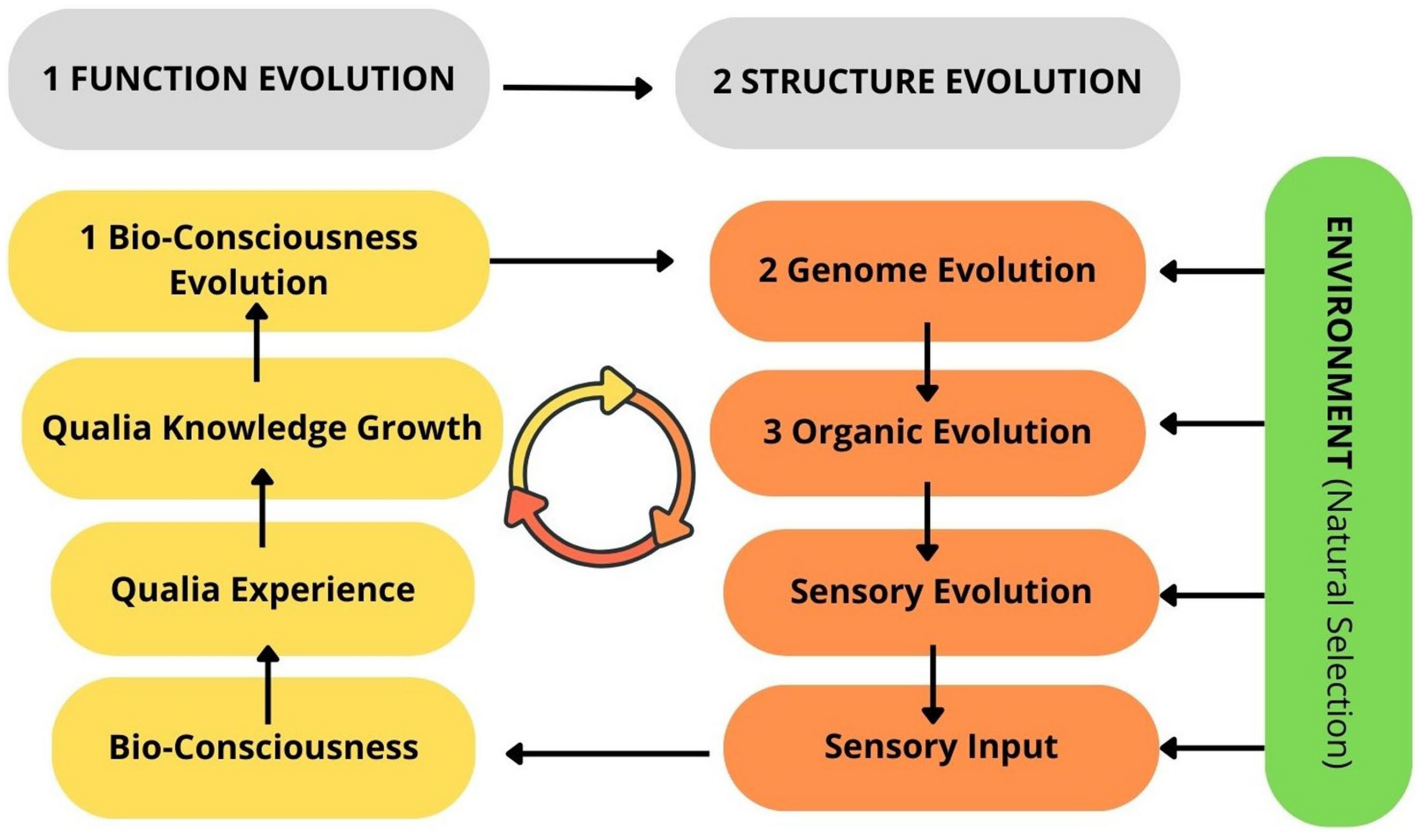
Popper’s theory of evolutionary hierarchy: (1) bioconsciousness evolution; (2) genome evolution; (3) organic evolution.

Popper summarized that preference plays a leading role in evolution (Popper, 2010, p. 172); thus, behavioural or functional changes are always first, followed by anatomical or structural changes, which are last, in a cyclical direction. This framework could be adapted to the evolution of as follows: first, bioconsciousness evolution leads to genome evolution, which in turn drives anatomical transformation (Figure 6). Moreover, this theory explained that evolution is a result of genome evolution (direction), not genome mutation (randomness); therefore, genome evolution is the result of bioconsciousness evolution. According to Popper, “*active Darwinism*” is when “organisms have agency and make choices” (Noble and Noble, 2021) and he concluded that “*all life is problem solving*; acquiring new knowledge is always a purposeful activity.” (Niemann, 2014, p. 90; Noble and Noble, 2021)

It is hoped that, based on these three theories presented, it can reasonably be concluded that the Absolute BioConsciousness and Relative Cell Biomatter (ABC) theory. proposes that (1) bioconsciousness evolution precedes (2) genome evolution and (3) organic evolution, in other words, *mind before matter, function before structure*.

#### 2.3 Universal Genome Evolution (UGE) theory

“Nothing in evolution makes sense except in the light of *genomics*.” (Shapiro, 2016)

“The most basic tenet of evolution science—*the genetic relatedness of all living organisms*—is abundantly supported by molecular biology, in particular the universal features of the triplet code for amino acids and the similarities of core cell structures, like the ribosome, associated translation factors, and DNA and RNA polymerases.” (Shapiro, 2016)

“Instead of the “constant genome,” subject to *accidental modification*, we know today that cells possess “*read–write genomes*” they can alter by numerous biochemical processes capable of rapidly restructuring cellular DNA molecules. Genomics has modernized our understanding of the evolutionary process. Rather than viewing genome evolution as a happenstance series of *copying errors*, we are now in a position to study it as a complex biological process of *active self-modification*.” (Shapiro, 2016)

“Evolution is merely a change in allele frequencies. There is *no other mandatory attribute to the evolutionary process*.” (Graur, 2016)

“According to this *DNA-centred perspective*, stable inherited changes in organismal properties result primarily from *alterations in the genome*” (Shapiro, 2016). This section will further explore the direct relationship between bioconsciousness and DNA molecules of every living cell. The Universal Genome Evolution (UGE) theory. posits that cell DNA is a *structural correlate* of cell bioconsciousness for the following reasons:

1) Universal structure.

“DNA usually consists of two antiparallel complementary strands twisted around each other to form a right-handed double helix. Each strand is a linear polynucleotide consisting of four kinds of nucleotides. There are two purines, adenine (A) and guanine (G), and two pyrimidines, thymine (T) and cytosine (C). A purine always pairs with a pyrimidine: A–T, G–C.” (Graur, 2016, pp. 5–6)

2) Universal genetic code.

“The correspondence between the codons (triplets of nucleotides) and the amino acids is determined by a set of rules called the genetic code. The genetic code for nuclear protein-coding genes is *universal*; the translation of almost all eukaryotic and prokaryotic nuclear genes is determined by *the same set of rules*. Since a codon consists of three nucleotides, and since there are four different types of nucleotides, there are 4^3^=64 possible codons.” (Graur, 2016, pp. 21–22)

3) One pathway of information flow.

“One pathway of information flow, usually worded as “*DNA makes RNA makes protein*.” In reality, the central dogma simply states that “once information has got into a protein it can’t get out again.” (Graur, 2016, p. 24)

4) Universal DNA replication.

“All organisms replicate their DNA before every cell division. During DNA replication, each of the two DNA strands serves as a template for the formation of a new strand. Each of the two daughters of a dividing cell inherits a double helix containing one “old” and one “new” strand.” (Graur, 2016, p. 8)

5) Hereditary information.

The word “gene” meant a unit of heredity, coined by Wilhelm Johannsen (Graur, 2016, p. 11). “The hereditary information of all living organisms is carried by DNA molecules. A gene is a sequence of genomic material (DNA or RNA) that is functional” (Graur, 2016, p. 11)

6) DNA is highly stable.

“DNA is a highly stable molecule in vivo, and it is usually replicated with extraordinary accuracy. Rare errors may occur. These errors are called mutations. Unfortunately, many mutations are deleterious.” (Graur, 2016, pp. 24–25)

7) Universal horizontal gene transfer.

“Horizontal gene transfer (also called lateral gene transfer) is defined as the movement of genetic information from one genome to another, specifically between two species. More recently, we have come to realize that horizontal gene transfer is extremely prevalent in evolution, such that *all organisms are in effect transgenics*.” (Graur, 2016, p. 431)

8) Universal vertical gene transfer.

“Vertical gene transfer, in which a parent passes genetic information on to its progeny.” (Graur, 2016, p. 431)

9) Universal transformation: antiquity.

“Transformation involves the uptake of histone-free or *naked DNA from the environment*. Transformation does not require a dedicated molecular or cellular vehicle to transport the genetic information from one organism to another. The simplicity of transformation is commonly regarded as a sign of *antiquity*. Hence, transformation is thought of as the oldest method of mixing DNA from different organisms, long predating the emergence of sex. *Transformation is nonspecific*; *any stray bit of DNA*, whether synthetic DNA or DNA derived from a long-dead animal, such as from a *mammoth bone*, can be incorporated into a *live bacterium genome*. (Graur, 2016, pp. 432–434)

10) The minimal genome for life.

“A cell that contains only components that are absolutely essential for life is called a minimal cell. A minimal cell has a *minimal genome* that contains only genes that are required for *survival*. In other words, the minimal genome is made of the smallest set of genes an organism needs to live. If the term “life” is comprehended solely from the perspective of the genetic material, then the minimal genome is the answer to the question “*What is life*?” (Graur, 2016, p. 463)

“The evolutionary search for the genome of the “smallest autonomous self-replicating entity,” which presumably would resemble the first cellular organism, was begun in the late 1950s” (Graur, 2016, p. 464). The search led to studies of mollicutes, a eubacterial taxon consisting of mycoplasmas, most of which are *distinguished by the absence of a cell wall*.” (Graur, 2016, p. 464)

“One day, a scientist will drop gene number 297 into a test tube, then number 298, then 299…and *presto*: what was not alive a moment ago *will be alive now*. For a number of reasons, this approach has not yet been successful, although great strides have been made in the field of genome synthesis.” (Graur, 2016, p. 464)

11) Gene essentiality.

“An *essential gene* is one that is critical for the survival of the organism carrying it and whose individual inactivation causes *inviability*.” (Graur, 2016, p. 466)

12) DNA nanotechnology & self-assembling molecules

DNA nanostructures as a programmable core building block: programmable self-assembling DNA nanostructures, autonomous molecular replication. (Lee et al., 2024)

13) DNA degradation during a cornification process

“The intracellular changes of cornification include three hallmarks of cell death and can be considered as the actual cell death process: 1) the molecular machinery that constitutes the cell’s ability to respond to stimuli from the environment is degraded, 2) the production of energy is stopped by the removal of mitochondria, and 3) the nucleus is dismantled and the DNA is destroyed.” (Eckhart et al., 2013).

“DNA origami, which uses DNA––the same biological building block that makes up the genome–– to create nano-sized structures onto which molecules can be positioned with nanometer precision.” (Douglas et al., 2025; https://bme.utoronto.ca/news/engineering-synthetic-immune-complexes-using-dna-nanotechnology/)

**Figure 7:**
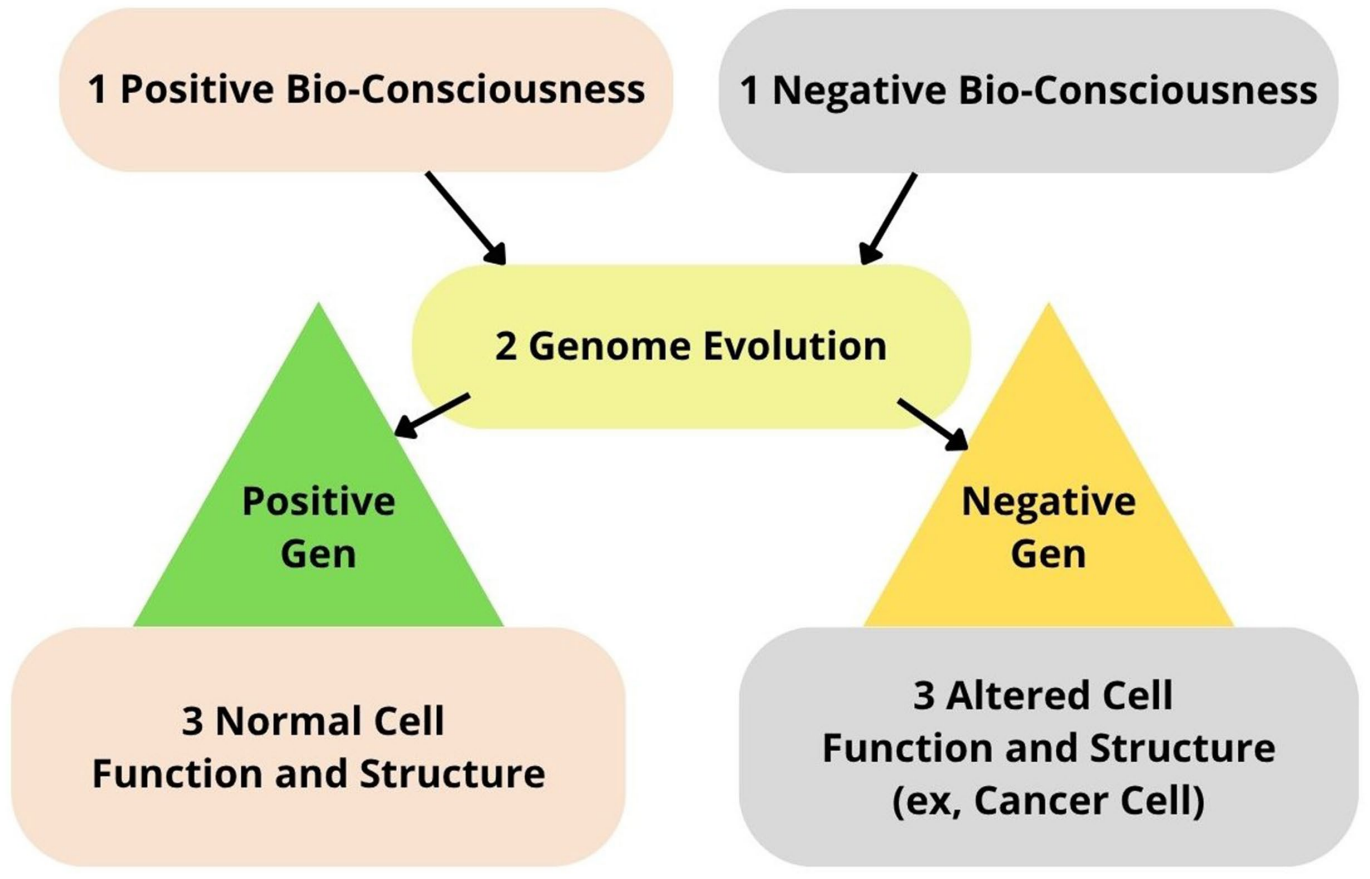
Universal Genome Evolution (UGE) theory. (1) bioconsciousness evolution (function); (2) genome evolution (biostructure).

The universal genome concept, as applied to all biological life, encompasses the following core principles: universal structure, universal genetic code, gene essentiality, universal DNA replication, universal horizontal and vertical gene transfer, and the universal transformation. The universality of the genome suggests that all life descended from a *single ancestral organism*, implying a *single universal cause*. Therefore, it could be reasonably concluded that *universal DNA is a structural correlate of universal bioconsciousness*. The idea that the external cell membrane could serve as a structural correlate of bioconsciousness is scientifically limited, since viruses and other living organisms, such as mollicutes, lack a true cell membrane. Additionally, “the RNA world hypothesis predicts that self-replicating RNAs evolved before DNA genomes and coded proteins” (Fine and Moses, 2025). Therefore, *self-replicating RNA molecules* highly probable a structural correlate of bioconsciousness before the emergence of *self-replicating DNA molecules* (Fine and Moses, 2025). However, the evolution of bioconsciousness (function) as a “primary thinking being” is always the primary cause of genome evolution (biostructure). Negative or altered bioconsciousness could be a *potential cause* of genetic mutations (Figure 7) (a hypothesis that requires further scientific investigation).

### 2.4 Ontogenetic Evolution of Universal Bioconsciousness (OEUB) theory

“Things which are the product of a gradual evolution–things which came from other things as unlike to them as oaks to acorns or chickens to eggs.” (Romanes, 1885, p. 54)

“A spermatozoon unites with an ovum, which we then designate as fertilized. A fertilized ovum is a complete cell which divides and continues dividing until the number of cells for the construction of an animal body has been produced. This number may be enormous. In the earliest stages of the embryo, the cells are remarkably like one another, but in the course of their further development, they become unlike or different. The development of simple cells into differentiated we call progressive development. In fact, *from the biological standpoint, we are really old by the time we are born*.” (Minot, 1913, pp. 12, 25, 33)

**Figure 8:**
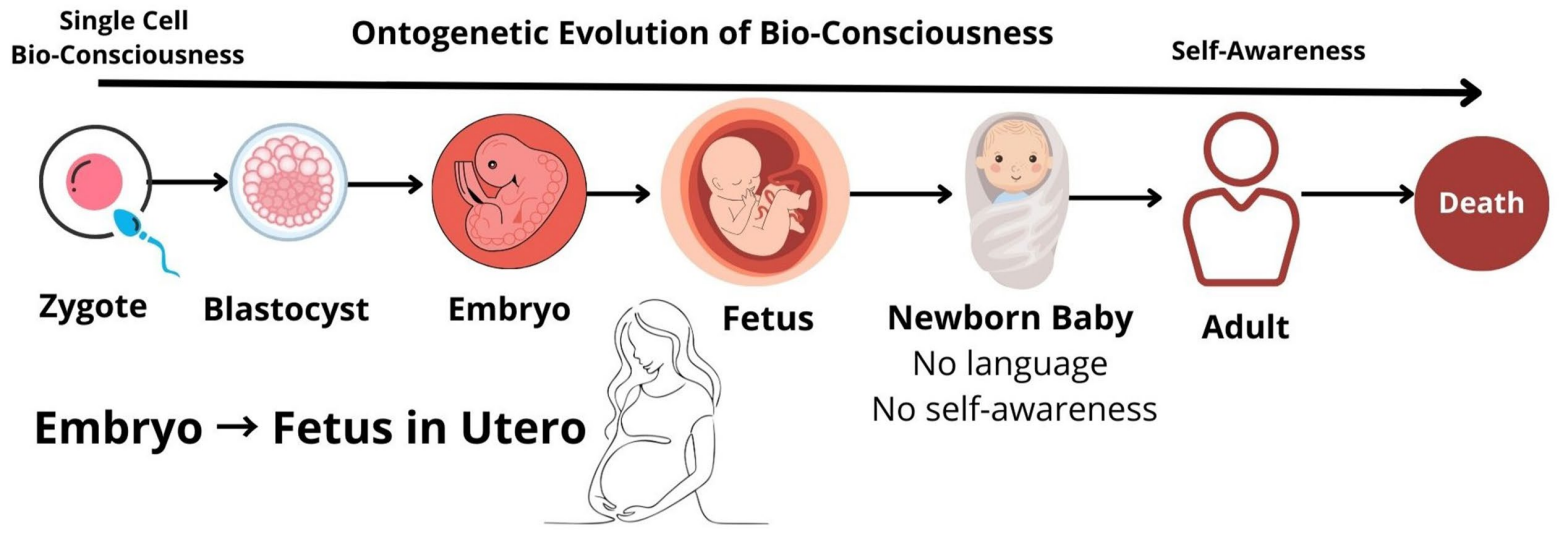
Ontogenetic Evolution of Universal Bioconsciousness (OEUB) Theory.

Universal BioConsciousness (UBC) theory or Bioconsciousness originates from Bioconsciousness (BoB) theory proposes that bioconsciousness, which originated from the first FUCA bioconsciousness, continues uninterrupted in all biological descendants. Ontogenetic Evolution of Universal BioConsciousness (OEUB) theory suggests that the development of bioconsciousness within an individual’s lifetime begins with the first fertilized egg cell, formed through sexual reproduction. In asexual reproduction, bioconsciousness evolves from the bioconsciousness of a pre-existing parent cell, which divides and gives rise to two genetically identical daughter cells, each with its own bioconsciousness. Therefore, in humans, life-consciousness starts from the first fertilized egg cell—zygote, which leads to further development of an embryo and then fetus inside the uterus of mother during 9 months of pregnancy; so, there is uninterrupted gradual evolution of life-consciousness from zygote to a newborn baby till death with progressive development of higher-order bioconsciousness and organic evolution (Figure 8). “I attempted to convince you …that which we called the condition of old age, is merely the culmination of changes which have been going on from the first stage of the germ up to the adult, the old man or woman.” (Minot, 1908).

Furthermore, Haeckel provides insight into the concept of “innate knowledge” in newborns, offering a perspective on how fundamental learning capabilities may be present from birth. For example, a wildebeest calf is able to rise and suckle and then run with the herd within minutes of birth.

**Figure 9:**
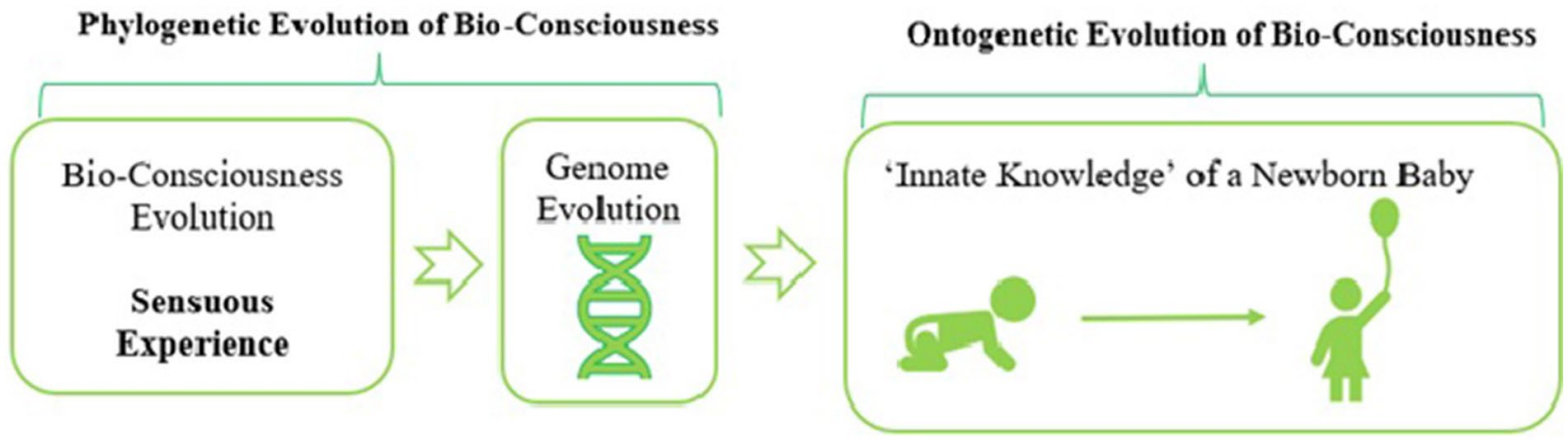
Haeckel’s theory of innate knowledge.

“All knowledge springs from sensuous perceptions. In opposition to this statement, the innate, a *priori knowledge* of man may be brought up; but we can see that the so-called a *priori knowledge* can, by Darwin’s theory, be proved to have been acquired a posteriori, being based on experience as its first cause. Knowledge which is based originally upon purely empirical observations, and which is therefore a *purely sensuous experience*, but has then been transmitted from generation to generation by inheritance, appears in later generations as if it were independent, innate, and a priori. In our late animal ancestors, all our so-called “*a priori knowledge*” was originally acquired a posteriori, and only gradually became a priori by inheritance. It is based in the first instance upon experiences, and by the laws of inheritance and adaptation, we can positively prove that *knowledge a priori* and *knowledge a posteriori* cannot rightly be placed *in opposition* as is usually done. On the contrary, sensuous experience is the original source of all knowledge. For this reason alone, all our knowledge is limited, and we can never apprehend the first causes of any phenomena.” (Haeckel, 1880, pp. 31–32; Figure 9)

From an ethical standpoint, any intervention against life from conception (the first fertilized cell) to the birth of a newborn baby should be critically re-examined in the light of new evidence.

### 2.5 Phylogenetic Evolution of Universal BioConsciousness (PEUB)

“In fact, the history of individual development, or ontogeny, is a short and quick recapitulation of paleontological development, or phylogeny.” (Haeckel, 1880, pp. 10–11)

In fact, the ontogeny of bio-consciousness—the individual evolution of bioconscious—is a condensed and accelerated recapitulation of the phylogeny of bio-consciousness—the ancestral evolution of cellular bioconsciousness across biological time. In other words, each organism’s bioconsciousness journey, beginning at the unicellular zygote, mirrors the evolutionary ascent of bioconsciousness from the first conscious cell (FUCA) through multicellular complexity. it is inherited and gradually elaborated through biological development: the zygote, a single fertilized cell, embodies the basic unit of bio-consciousness, serving as the structural and informational seed from which higher levels of consciousness emerge, such as complex human cognition. BioConsciousness Evolution Laws or Principles: 1. Structural dependency: Higher-order bioconsciousness requires a biological substrate capable of supporting it. The unicellular state provides the minimal viable structure for consciousness to exist and evolve. 2. Unicellular origin of multicellular life. BioConsciousness evolves over time through biological development. It cannot be instantaneously instantiated at a multicellular level without passing through the unicellular phase. A comprehensive understanding of the evolutionary history of bioconsciousness will help us understand its role in our life, future prognosis, and discover new scientific insights. Moreover, in the era of rapid technological advancement, such as artificial intelligence, phylogenetic classification will guide us in developing safe and responsible algorithms and avoiding their unintentional, harmful effects on the evolution of bioconsciousness.

The modern evolutionary basis of classification is grounded in *biological classification*: organisms are grouped together based on anatomical similarities and differences to identify a biological category, such as species, genera, families, orders, or classes (Richards, 2016, pp. 1–3). Species is the primary biological classification and the fundamental unit of both taxonomy and evolution (Richards, 2016, pp. 1–3). Considering recent discoveries of the primary role of bioconsciousness in the evolutionary process, we need a hierarchical and comprehensive framework for classifying living things based on the evolution of bioconsciousness. According to Richards, there are four questions arising: (1) theoretical, what should a classification represent? (2) Operational, how should a classification be generated? (3) The role of tree thinking, the challenges of the tree metaphor about evolution; (4) ranking, is it possible to modify the Linnaean hierarchy and naming system?(Richards, 2016, pp. 8–9). The Phylogenetic Evolution of Universal BioConsciousness (PEUB) is ultimately shaped by the cumulative evolutionary changes that unfold throughout its ontogenetic development. The evolution of UBC occurs within an organism’s lifetime, with these evolutionary changes then being passed down to offspring through hereditary mechanisms, such as Universal Genome Evolution (UGE). *There is no universally established distinction between the different stages of UBC evolution*. However, the development of the nervous system and the progression of mental faculties could serve as a valuable marker of the Phylogenetic Evolution of Universal Bioconsciousness (PEUB)

### 2.6 Anesthesia, Near-Death Experience, and Non/Unconscious states

In the article “What we (can) know about consciousness,” the author discussed her experience of the levels of consciousness after the recovery from anesthesia: “My awakening from anesthesia as a prime example of going from 0 to 100” (Uddin, 2025). In clinical practice, general anesthesia is designed to eliminate self-awareness or higher-order consciousness, and it is considered a “level 0” state in clinical terms. However, there is a philosophical question that touches on both the epistemology and the ontology of consciousness science: How can we know that the gradual return to self-awareness (a higher-order of consciousness) from general anesthesia after surgery can be ranked from 0 to 100? What does it mean for it to “return” or “increase”? Where does self-awareness go during a surgical operation under general anesthesia? Is the idea of ‘complete non or unconscious’ states ontologically meaningful? If we apply Descartes’ principle that “it is impossible the same thing can at once be and not be” (Descartes, 1965, p. 184), then the following question arises: Can higher-order consciousness or self-awareness be entirely absent (i.e., at level 0) during general anesthesia, and then fully return (i.e., to level 100) after recovery—preserving its continuity and memory, such as recalling the article deadline as before the surgery? If we accept Descartes’ axiom that “it is impossible the same thing (same self-awareness, added by author) can at once be and not be,” then the notion that ‘same self-awareness’ disappears entirely (level 0) during anesthesia and then after a couple of hours returns fully (level 100) raises a paradox: If self-awareness truly ceased to exist, then what returns after anesthesia would not be the ‘same self-awareness’—it would be a new instantiation, not a continuation. Yet, patients often recall pre-anesthesia intentions (e.g., remembering an article deadline), suggesting a continuity of identity and memory. This implies that self-awareness cannot be at level 0; rather, it is temporarily suspended or rendered inaccessible, preserving its ontological substrate. Thus, the conclusion becomes: Self-awareness cannot simultaneously be and not be. Therefore, if it returns with continuity, it must not have ceased to exist, but instead persisted in a latent or non-manifest state during anesthesia. Thus, the term ‘unconscious’ or ‘non-conscious’ states (level 0) may be ontologically misleading. A more accurate framing would be: ‘latent state of higher-order bioconsciousness or latent state of self-awareness. This perspective is further supported by ABC Theory, which posits that bioconsciousness is independent of biomatter. Furthermore, this theory also supports the notion that bioconsciousness may persist beyond physical death. For example, this view raises several questions about near-death experiences (NDEs), in which individuals report out-of-body experiences during episodes of clinical death (Greyson and Pehlivanova, 2025), which ontologically challenge the materialist views of present consciousness science. Therefore, in light of new evidence, reconsidering the NDEs, non/unconscious states in clinical practice, and death is important to fully understand the ontological nature of bioconsciousness and its epistemological implications.

## 3 Part 2: Search after Truth: theory of Descartes “Cogito Ergo Sum”

### 3.1 Descartes’ First Principle: Bio-Consciousness as a Primary “Thinking Being”

Robinson et al. (2024) proposes agreement on key definitions on “*consciousness*” and “*cognition*” terminology as follows: “*cognition*” is “the ability to receive, process, store and act on information from the environment, including memory, learning and making decisions,” and “*consciousness*” as “the capacity to have feelings or experiences, yielding a subjective or first-person point of view” (Robinson et al., 2024). To whom does “*cognition*” belong? In the following pages, I will try to answer this question.

**Figure 10:**
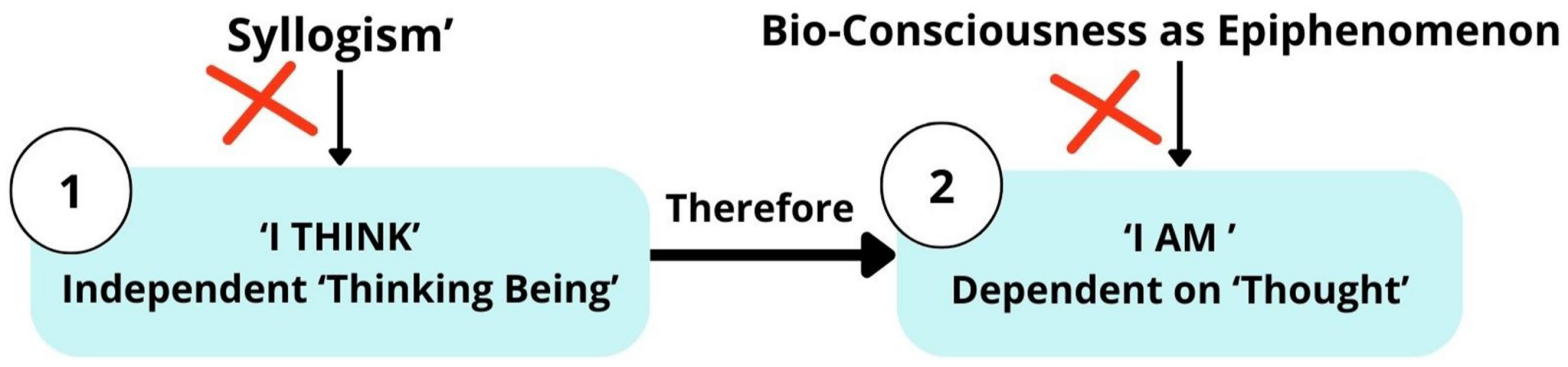
Syllogism “Cogito ergo sum.” Major premise: everything which thinks exists; Minor premise: I am thinking; conclusion: therefore, I exist.

Descartes stated: “*I think, hence I am*…accept it as the *first principle of the philosophy*” (Descartes, 1965, p. 27). The “I think” concept needs to be further clarified: Do we need to know it “*immediately* and directly, or only *mediately*, via some process of *thinking*”? (Descartes, 1976, p. xx) Descartes stated:

I observed that in the words “*I think, hence I am*”…, I *see very clearly* that in order to *think* it is necessary to *exist*, I concluded…that all things… are true only observing (Descartes, 1637, p. 76). I am—I exist: this is certain; I am conscious that I exist (Descartes, 1965, pp. 86–88). When we take note of the fact that we are thinking beings, this is a *primary notion*, which is not derived from any syllogism. When someone says, “*I think, therefore I am*, or I exist,” they do not deduce existence from “*thought*” but recognize it as if it were a thing known *per se* (by itself). If they were deducing it by means of a *syllogism*, they would have to know previously the major premise “Everything which thinks is, or exists,” that it is *impossible* that they should *think without existing* (without bioconsciousness, added by author) (Figure 10) (Descartes, 1976, p. 4, p. xx). It is a “thinking thing,” its nature or essence consists only in its being a thing which “thinks.” I clearly apprehend nothing, except that I was a “thinking thing,” or a thing possessing in itself the “faculty of thinking.” Nothing besides thinking belongs to the essence of the mind (Descartes, 1965, pp. 71–72). All that I have, up to this moment, I received either from or through the senses … from sight, by which I have perceived colours, shapes…through hearing… (Descartes, 1965, p. 80; Descartes, 1976, p. 3). In view of this, I do not doubt that the mind begins to think as soon as it is implanted in the body of an infant (Descartes, 1976, p. 8).

**Figure 11:**
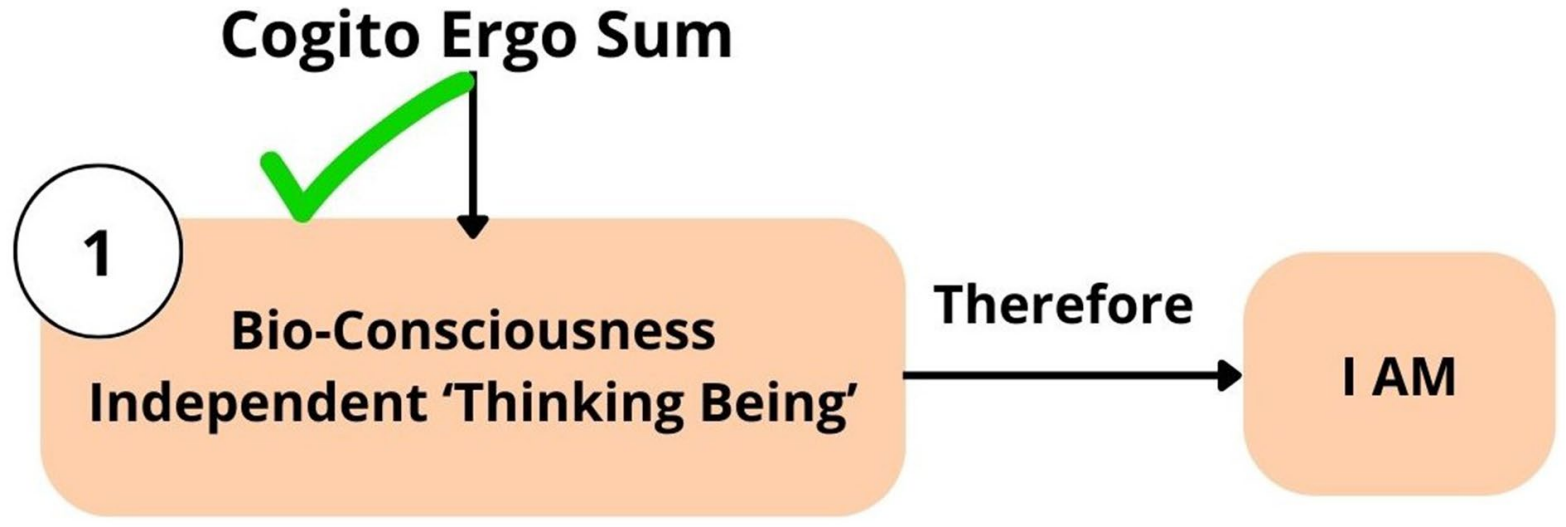
SeArch afterTruth: Descartes’ theory “Cogito ergo sum.” “We are thinking beings, this is a primary notion” (Descartes, 1976, p. xx).

According to Descartes, the principal attribute of existence is the capacity for thought. Descartes’ definition of “thought” is simply a synonym of “consciousness” (McRae, 1972, p. 55). “*Consciousness*” is the best concept to convey the whole meaning of Descartes’ “*thought*” (McRae, 1972, p. 56). Consciousness in Descartes’ philosophy is not a verbal thought or dependent on verbal thought, but is described as independent “*conscious sensory experience*,” like feeling, hearing, seeing, or “qualia” *experience* (McRae, 1972, p. 56). In other words, Descartes’ term “*thought*” is “everything that we are *immediately conscious of*” (McRae, 1972, p. 59). In the seventeenth century, the term “*cogitare*” was translated into English as “*to be conscious of*” (McRae, 1972, p. 56). To conclude, in the phrase “Cogito ergo sum,” the term “*Cogito*” represents “*consciousness*” as a thinking being *per se* (by itself), existing independently of “verbal thought” (Figure 11). According to Descartes’ philosophy, “*cognition*” and “*consciousness*” are the same concepts, and *consciousness is* “*cognition*” or “thinking being” itself (*per se*), which answers the question “to whom does ‘*cognition*’ belong?” Although it is challenging to define the exact meaning of the term “bioconsciousness,” a clear definition is necessary, acknowledging all previous scientific knowledge (Box 2).

BOX 2 Definition of bioConsciousness

To avoid misunderstanding and clarify the precise meaning of the term “ bioconsciousness,” we can establish a definition using the following five methods: (1) classification, (2) synonym, (3) example or illustration, (4) enumeration, (5) function (Martin and Kroitor, 1980, p. 127).

- *Definition by classification* or Aristotelian method involves only two steps: (1) “place the word in its family (genus), and (2) differentiate the word from all others in its family” (Martin and Kroitor, 1980, p. 127).

- Bio-consciousness belongs to one family of earth biological life, as bioconsciousness originates from pre-existing bioconsciousness; thus, its nature is universal (one origin).
- Bio-consciousness is “absolute”—“independent, causal, universal, unitary.”

II *Definition by synonyms* uses different words with the same meaning that can be easily understood (Martin and Kroitor, 1980, p. 129).

- Bio-consciousness encompasses the mind, cognition, thinking as a process, first-person “qualia” experience, subjective experience, memory, will (or purpose), and is coterminous with biological life.
- Therefore, the definition of life is “Life is positive bioconsciousness.”

III *Definition by example* or illustration.

- “Imagine the taste of a ripe strawberry…When we speak of consciousness, we are referring to the very fact of experience” (Cooke, 2024, positive).

IV *Definition by enumeration* is “listing the members of the class represented by that word” (Martin and Kroitor, 1980, p. 131).

- Bio-consciousness encompasses both positive and negative thinking thing.

V *Definition by function*—operational definition explains “how something operates or how it works” (Martin and Kroitor, 1980, p. 131).

- “The function of consciousness is to dislocate in time the reactions from sensations.” Purpose-driven (Minot, 1902, p. 4).
- “It is a ‘thinking thing;’ its nature or essence consists only in its being a thing which thinks. I clearly apprehend nothing, except that I was a ‘thinking thing,’ or a thing possessing in itself the faculty of thinking. Nothing besides thinking belongs to the essence of the mind.” (Descartes, 1965, pp. 71–72).
- Bio-consciousness comes in “unicellular-unit” (primary consciousness) and “multicellular-unit” (a higher order of evolutionary development of BC), where everyone possesses their own subjective “qualia” experience, representing a distinct form of private or first-person conscious knowledge, yet existing at different stages of progressive evolutionary development.

*The one-sentence definition*: Bio-consciousness is an absolute, universal “thinking” living being, with both positive and negative states, and causal force from which bio-matter emerges as a secondary and dependent phenomenon; it has a unit-based nature (unicellular or multicellular). BioConsciousness undergoes a self-sustaining process of self-replication by passing the hereditary information (DNA/RNA) by cell division process (1BC becomes 2BC), progressing from primary cellular bioconsciousness to complex multicellular bioconsciousness, including self-awareness in human beings, which manifests in the gradual evolution of biological complexity, and cell biomatter is secondary and the genome (DNA/RNA) is a structural correlate of bioconsciousness.

*Definition of Life*: Life is a self-replicating, self-sustaining positive bioconsciousness that undergoes continuous cognitive and biological evolution through sensory interaction with its environment, using genome evolution as a structural correlate for the transmission of hereditary information.

### 3.2 Verbal Communication Sense (VCS) theory

“In every sense, there is passivity which indicates dependency.” (Descartes, 1965, p. 174)

“Consciousness is not the voice in your head…Imagine the taste of a ripe strawberry…*Consciousness does not require thought* or the ability to self-reflect. When we speak of consciousness, we are speaking of the very fact of *experience*.” (Cooke, 2024, p. viii)

“What *thought (cogitatio)* is. By the word ‘thought,’ I understand all that which so takes place in us that we of ourselves are *immediately conscious of it*; and, accordingly, not only to understand (intelligere, entendre), to will (velle), to imagine (imaginari), but even to perceive (sentire, sentir), are here the same as to ‘*think*’ (cogitare, penser).” (Descartes, 1965, p. 167)

This section will try to answer the following question: How does Descartes’ first principle, “Cogito ergo sum,” explain the relationships between bioconsciousness, verbal thought, and verbal language? At the beginning of unicellular life, there were no words or verbal thoughts, but sensing was an elementary form of intelligent response (Damasio, 2021, pp. 13–14). Therefore, verbal thought and verbal language are the *latest inventions* of evolution, but they have a long evolutionary history (Darwin, 2010, p. 78; Pinker, 1995). Darwin, in his paper “*Descent of Man*” (1871), described communication as a sociability function: animals that first recognize an enemy warn others by making danger voice signals (Darwin, 2010, p. 78). As mutual dependence for survival and helping each other in dangerous situations requires strong communication skills, danger voice signals could be an early stage in the development of a verbal communication system (Darwin, 2010, p. 78).

**Figure 12:**
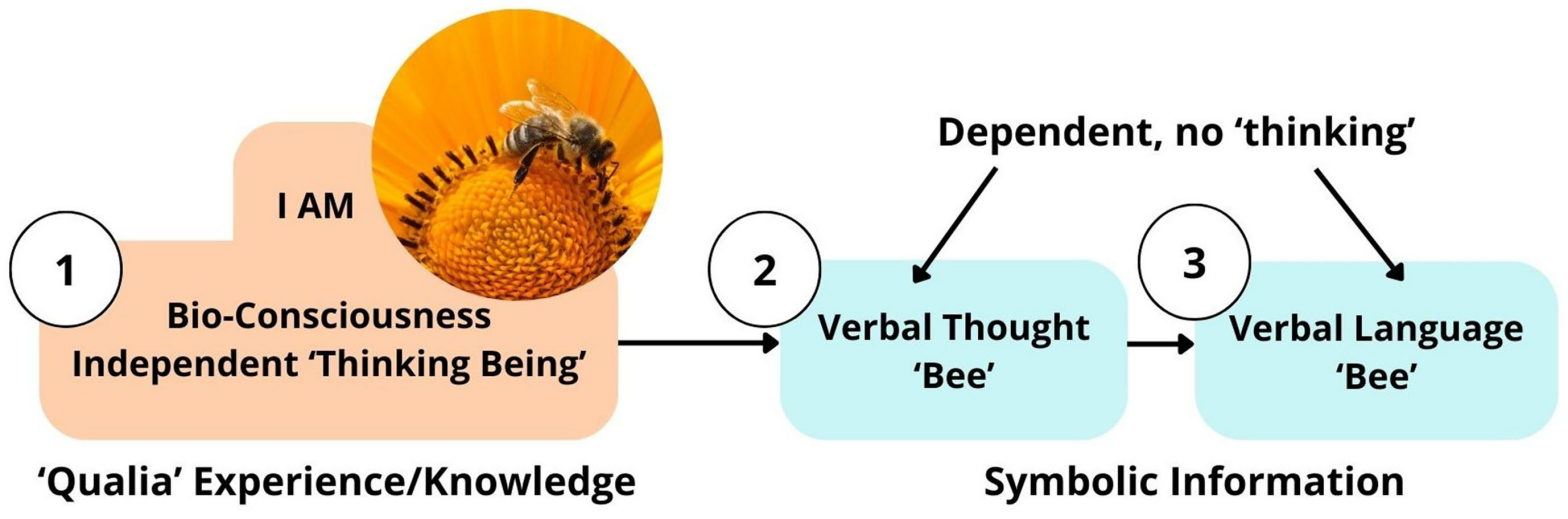
Symbolic (Verbal) Learning (SL) Theory: (1) foreign verbal language (unfamiliar symbolic information); (2) unfamiliar verbal thought; (3) no BioConsciousness development and evolution (no knowledge and understanding growth). VCS - verbal communication sense.

In the previous section, we have concluded that bioconsciousness (“cogito”) is a thinking substance as a *primary notion*, which has the ‘*faculty of thinking*.’ “It is impossible the same thing can at once be and not be” (Descartes, 1965, p. 184); then it is impossible that ‘verbal thought’ can at once ‘think’ and ‘not think.’ If consciousness is a “thinking being,” what is the function of “verbal thought”? Does “verbal thought” have the faculty of “*pure thinking*” without consciousness? Descartes replied: “He who thinks must exist while he thinks, *it is impossible that they should think without existing* (without bioconsciousness, added by author)” (Descartes, 1965, p. 184; Descartes, 1976, p. xx); Descartes distinguishes two types of things: “*absolute*,” which is independent, causal, universal, and “*relational*,” which means dependent on absolute and resultant (Box 1); thus, “verbal thought” is inherently dependent and resultant on bioconsciousness, which is absolute, causal, and independent. Further, Descartes states: “All the things we perceived by our senses had an existence beyond our thought” (Descartes, 1965, p. 192). *It is evidence of the dependency of verbal thought on consciousness*: the latter “verbal thought” cannot be known apart from the former “ bioconsciousness.” Verbal thought is not an independent “thinking concept;” it is dependent on bioconsciousness; thus, we are “thinking” by consciousness, not by verbal thought. Therefore, “verbal thought” is (1) dependent on bioconsciousness, (2) passive, and (3) does not have ‘faculty of thinking,’ whereas consciousness is an active, independent ‘thinking faculty.’ If verbal thought, by itself, does not think, Minot’s question will state: “What is the essential function that ‘verbal thought’ performs?” (Minot, 1902, p. 4). In conclusion, the Verbal Communication Sense (VCS) theory states that verbal thought and verbal language serve as a symbolic communication “sense” that enables the transfer of qualia—both subjective experiences and knowledge (memory)—between individual bioconsciousness, relying on a shared understanding of *symbolic information*. There is an order or sequence of these three processes: (1) bioconsciousness—“cogito;” (2) verbal thought: communication “sense” or linguistic tool expressed in words; (3) verbal language: conveying words outwardly (spoken; written) (Figure 12). To conclude, “verbal thought—verbal language” could be considered as a *verbal communication sense system*.Buddhism also acknowledges the ‘mind’ (thoughts or ideas) as a sixth sense in the following quote:

“The second is the Aggregate of Sensations. In this group are included all our sensations, pleasant or unpleasant or neutral, experienced through the contact of physical and mental organs with the external world. They are of six kinds: the sensations experienced through the contact of the eye with visible forms, ear with sounds, nose with odour, tongue with taste, body with tangible objects, and mind (which is the sixth faculty in Buddhist Philosophy) with mind-objects or thoughts or ideas. A word about what is meant by the term ‘Mind’ (manas) in Buddhist philosophy may be useful here. Mind is only a faculty or organ like the eye or the ear. It can be controlled and developed like any other faculty… The difference between the eye and the mind as faculties is that the former senses the world of colours and visible forms, while the latter senses the world of ideas and thoughts and mental objects. We experience different fields of the world with different senses. We cannot hear colours, but we can see them. Nor can we see sounds, but we can hear them. What of ideas and thoughts? They are also a part of the world. But they cannot be sensed, they cannot be conceived by the faculty of the eye, ear, nose, tongue or body. Yet they can be conceived by another faculty, which is ‘mind.’ Hence, mind (manas) is considered a sense faculty or organ, like the eye or the ear.” (Rahula, 1959, pp. 21-22)

“The fifth is the Aggregate of Consciousness. Consciousness is a reaction or response which has one of the six faculties (eye, ear, nose, tongue, body and mind) as its basis... For instance, visual consciousness has the eye as its basis and a visible form as its object. Mental consciousness has the mind (manas) as its basis and a mental object, i.e., an idea or thought, as its object. So, consciousness is connected with other faculties.” (Rahula, 1959, p. 23)

### 3.3 Positive and negative BioConsciousness theory

“Out of the same mouth come praise and cursing. Can both fresh water and salt water flow from the same spring?” Bible, James 3: 10–11.

It is well known that human beings have positive *thought thoughts* *negative thoughts*.In the previous section, we concluded that verbal thought is a “verbal communication sense system,” dependent on bioconsciousness, and does not possess its own “thinking faculty.” As a result, we “think” by bioconsciousness and verbal thought is a linguistic device/sense, which translates meaning into words and spoken language. Therefore, the concept “negative thought” is meaningless, as verbal thought is passive and dependent on bioconsciousness. So, “*it is impossible the same thing can at once be positive and negative*” (Descartes, 1965, p. 184);

I am arguing, then, that there are two distinguishable states of bioconsciousness: “positive” and “negative” bioconsciousness. The effect of these states is expressed from the unicellular level (primary bioconsciousness) to the multicellular level (higher order of bioconsciousness, or self-awareness). Furthermore, I suggest that emotions could play a crucial role in understanding and distinguishing between the two types of bioconsciousness, as negative emotions, such as anger, fear, and hatred, can shape bioconsciousness in one way, while positive emotions like love, compassion, and patience contribute to a more positive experience of bioconsciousness. Although this theory needs further scientific investigation, it has broad implications for the origins of biological and psychological diseases in shaping health outcomes. For instance, for psychiatric (mental) disorders, negative thoughts may be symptomatic of a “negative” state of bioconsciousness, potentially serving as a primary factor in the development of mental illnesses, which negatively affects the higher order of bioconsciousness evolution, such as self-awareness. This initial concept requires further scientific investigation.

## 4 Ethics of artificial intelligence

“Without sufficient caution, we may irreversibly lose control of autonomous AI systems, rendering human intervention ineffective. Large-scale cybercrime, social manipulation, and other highlighted harms could then escalate rapidly. This unchecked AI advancement could culminate in a large-scale loss of life and the biosphere, and the marginalization or even extinction of humanity.” (Bengio et al., 2024)

In the consensus paper “*Managing AI risks in an era of rapid progress*,” the Godfather of AI and Nobel laureate, Geoffrey Hinton, Yuval Noah Harari, the best-selling author of the books “Nexus” & “Human Sapiens,” and Global AI Safety Initiative founder and advocate Yoshua Bengio warn the global community about the novel risks to humanity posed by AI’s rapid progress, which can exacerbate global injustice and inequity (Bengio et al., 2024). Thus, the ethics of artificial intelligence need to be carefully analyzed in the context of the new research evidence outlined in this paper.

### 4.1 Implications for Artificial Consciousness

“AI systems can learn from experience and perform human-like tasks, and they may be considered more intelligent than humans based on many metrics. However, no matter its manifest powers, AI systems cannot be conscious because they never experience internal doubt or have any internal, subjective state of preference.” (Miller et al., 2025)

“Since all conscious beings are made of cognitive cells, consciousness at whatever scale is judged appropriate is necessarily a cell-based phenomenon. Therefore, understanding the obligate conditions of cellular cognition are highly pertinent to considering the possibility of consciousness in AI machines.” (Miller et al., 2025)

“Consciousness is the self-referential cell-based capacity to apprehend informational uncertainty within the context of a sufficient biological architecture, permitting continuous adjustments as problem-solving”. Machines and AI technology are not conscious since they do not experience doubt and have no internal states.” (Miller et al., 2025)

The Association for Mathematical Consciousness Science (AMCS) published an *Open Letter* on the responsible development of AI, asking for the acceleration of consciousness research (Association for Mathematical Consciousness Science, 2023). The primary concern of the letter is whether human-level consciousness will emerge with the increasing computational power of AI technologies and whether they will further align with human values (Association for Mathematical Consciousness Science, 2023). The ethical development and implementation of artificial intelligence is the most pressing challenge in modern society, and indeed, an urgent answer is needed. Furthermore, social media authors are increasingly disseminating the idea: “Could AI become conscious?” (Glover, 2025a, 2025b). Considering the findings of this paper, if consciousness is tied to biological lineage and originates from pre-existing consciousness, then the possibility of emerging artificial consciousness is close to zero. Theoretically, according to the BioConsciousness originates from BioConsciousness (BoB) theory life- bioconsciousness cannot emerge spontaneously from non-living bodies, instead bioconsciousness can only emerge from the pre-existing bioconsciousness of a living being.

This idea that computers will not possess human-like bioconsciousness was also outlined in the paper “*Biological mechanisms contradict AI consciousness*” (Miller et al., 2025), where the authors conclude that “biological consciousness is cell-based, and lacking interior sensibilities, AI computers will not become conscious. Accordingly, machines and AI systems are neither alive nor conscious.” Therefore, AI development and implementation should be under the control and responsibility of human experts, particularly autonomous AI agent systems. This is especially important in critical areas, such as healthcare and its implementation among vulnerable populations like children and elderly individuals. AI-generated fake content, deepfake, AI cyber risk, risks from malfunctions, AI bias, “loss of control” scenarios, unexplainable “black box” AI models, AI hallucinations, privacy violations, and risks from malicious use could be a serious threat to the global community (International AI Safety Report, 2025). “General-purpose AI is already causing harm today due to malicious use and malfunctioning, for instance through deepfakes, scams and biased outputs” (International AI Safety Report, 2025).

In contrast, the integrated information theory (IIT) (Tononi, 2004; Oizumi et al., 2014) argues that consciousness is intrinsic to any system that integrates information beyond a certain level, regardless of its substrate; as a result, consciousness might emerge in artificial systems under sufficient complexity.

“One of the central postulates of IIT is what Tononi refers to as “phi,” or symbolically designated as “Φ.” According to IIT, phi is a measure of “*integrated information*.” According to some interpretations of the concept of phi, *virtually anything* with interacting parts that has a phi value *above 0* is therefore “*conscious*.” So, by this line of reasoning, consciousness could be ascribed to simple, *inanimate* entities like *thermostats* or even further down to a *single proton* with its interacting quarks. It has been considered by many critics from various fields as endorsing a variety of *panpsychism*.” (Feinberg, 2025)

“So, in contrast to the postulates of IIT, I argue that the *substrate of living things* is also likely to be an *essential prerequisite* for the emergence of sentience ( bioconsciousness, added by author). Computers with advanced AI capabilities are very complex with *astronomically high phi values* via *IIT criteria*—or for that matter complex via any criteria—but they are neither *alive nor sentient*. There is the *strong possibility* that the substrate that enables the emergence of sentience very well might specifically *require the biological features* that enable the emergence of life in addition to complex neurobiological features.” (Feinberg, 2025)

“IIT fails to take into account the *critical role that the substrate* of the parts of a system play in the emergence of sentience. By this account, IIT does not solve the many of biological, neurobiological, evolutionary, and philosophical issues.” (Feinberg, 2025)

Although this paper supports the notion that human-like bioconsciousness cannot emerge from non-living entities, I acknowledge that this area needs further scientific investigation by defining the clear distinctions and underlying mechanisms between machine learning (symbolic information) and human cognition ( bioconsciousness: qualia knowledge/experience).

### 4.2 Implications of Verbal Communication Vense theory

“Evolution cannot create something out of nothing.” Graur, 2016, p. 2)

Scientists are working on creating machines that possess human-like intelligence, including a recent study that evaluated large language models (LLM) for theory of mind (ToM) tasks, hypothesizing “it may emerge as a by-product of AI’s training to achieve other goals” (Kosinski, 2024). The verbal communication sense (VCS) theory posits that verbal thought and verbal language are the “verbal communication sense system,” which lacks its own independent thinking faculty and is dependent on bioconsciousness. Based on this new evidence, a following definition of AI have been proposed (Box 3).

Implications of Verbal Communication Sense Theory on Education and Future Learning (title of the section).

Understanding the relationship between bioconsciousness evolution and education is critical (Stoltz et al., 2024). Moreover, the importance of quality education is even more underscored by the rapid development of AI. Therefore, we need a comprehensive theory of learning based on the new evidence regarding the Universal BioConsciousness (UBC) theory and Verbal Communication Sense (VCS) theory. Based on these theories, education needs to shift from the symbolic transmission of information to the evolution of knowledge and understanding; in other words, learning should be a process of bioconsciousness evolution, not just the absorption of symbols. According to VCS theory, all symbolic verbal information depends on bioconsciousness, so symbols are only meaningful through high-quality engagement with the student’s bioconsciousness. In this view, learning is not reproducing (memorizing) verbal symbols without understanding, but bioconsciousness evolution by expanding ‘qualia’ knowledge and experience. Fig. 13 explains the Symbolic Verbal Learning (SVL) theory, which states that merely the transmission of symbolic verbal information, without bioconscious involvement, does not lead to learning. Therefore, translating unfamiliar symbolic information without students’ bioconsciousness involvement is meaningless, and we must shift from teaching “what to know” toward cultivating “how to become.” In this perspective, LLM is a non-biological system without bioconsciousness (no understanding) that imitates human verbal communication sense (VCS): verbal thought and verbal language.

In this perspective, LLM is a non-biological system without bioconsciousness (no understanding) that imitates human verbal communication sense (VCS): verbal thought and verbal language.

LLMs and the Mimicry of Communication

Large Language Models (LLMs) simulate communication by predicting symbolic patterns rather than by experiencing meaning, as they lack bioconsciousness-based learning (Mannekote et al., 2025). Symbolic learning involves processing information through language, numbers, and visual symbols. If students rely solely on “symbolic learning” - memorization and/or solving intellectual problems using ChatGPT without a deep understanding rooted in bioconsciousness development - they risk developing a fragmented understanding. In other words, symbolic learning (SL) emphasizes representation without proper understanding, while bioconsciousness-based learning (BCL) engages developing cognitive and critical thinking skills, thereby fostering the evolution of bioconsciousness. A paradigm shift in education is essential, as overreliance on artificial intelligence for cognitive tasks without bioconsciousness evolution will have serious consequences for human learning and development. Therefore, based on Symbolic Learning (SL) Theory, education needs to shift from solely relying on symbolic learning to multisensory engagement and experiential learning, and to encourage deep understanding through reflection and dialogue.

In the age of AI, we need to shift to the fifth way of science and education (Descartes, 1976, Figures 4, 5).

BOX 3 Definition of Artificial Intelligence Artificial Intelligence (AI) is a non-biological system of functional simulation that mimics the vision, hearing, smell, communication verbal sense (thought and language) without possessing bioconsciousness, through pattern recognition, data processing, and algorithmic training. It lacks phenomenological experience, intrinsic meaning-making, and is fundamentally distinct from the biologically grounded consciousness. From an ethical and legal perspective, AI does not qualify for human-related rights due to its lack of bioconsciousness and moral agency.

## 5 Conclusion

All living beings possess bioconsciousness, which is a vital force and essence of life; thus, life and bioconsciousness are intrinsically linked. Universal BioConsciousness (UBC) theory or BioConsciousness originates from BioConsciousness (BoB) theory posits that bioconsciousness emerges from pre-existing bioconsciousness, just as life originates from life. Absolute BioConsciousness and Relative Cell Biomatter (ABC) theory suggests that the evolution of bioconsciousness shapes the organic evolution, a concept also highlighted in Spencer’s theory of life, which posits that life precedes biomatter. Furthermore, Minot’s Theory of Biological Consciousness and Popper’s Theory of Evolutionary Hierarchy state that bioconsciousness is a primary driver of the evolutionary process, which precedes genome evolution and organic evolution. Universal Genome Evolution (UGE) theory states that RNA/DNA is a *structural correlate* of Earth’s Universal BioConsciusness The Ontogenetic Evolution of Universal BioConsciousness suggests that the zygote is the starting point of individual bioconsciousness evolution. Furthermore, Descartes’ theory “Cogito ergo sum” states that consciousness is *cogito* which implies a thinking thing by *per se*. According to Verbal Communication Sense (VCS) theory, verbal thought and verbal language are a “verbal communication sense apparatus” that lacks its own independent thinking faculty. Considering this evidence, artificial intelligence is highly unlikely to possess human-like bioconsciousness. Further research is needed to define transition markers for the Phylogenetic Classification of Universal Earth, Bioconsciousness Evolution.
